# Editorial: Endocrine imbalances of mineral ions and vitamins in chronic disease pathogenesis

**DOI:** 10.3389/fendo.2025.1667610

**Published:** 2025-08-29

**Authors:** Mor-Li Hartman, Hosam G. Abdelhady, Mohammed S. Razzaque

**Affiliations:** ^1^ The ADA Forsyth Institute, Cambridge, MA, United States; ^2^ College of Osteopathic Medicine, Sam Houston State University, Conroe, TX, United States; ^3^ University Kuala Lumpur Royal College of Medicine Perak (UniKL RCMP), Ipoh, Perak, Malaysia; ^4^ Department of Medical Education, School of Medicine, University of Texas Rio Grande Valley (UTRGV), Edinburg, TX, United States

**Keywords:** magnesium, vitamin D, klotho, niacin, selenium, X-linked hypophosphatemia (XLH), phenylketonuria (PKU)

Chronic diseases represent a major global health burden, accounting for nearly two-thirds of all deaths worldwide (1). In the United States, poor dietary habits and insufficient physical activity, both strongly associated with obesity, are leading contributors to this burden (1). Among the many factors implicated in the onset and progression of chronic diseases, disruptions in mineral and vitamin homeostasis play a critical yet often underappreciated role. Essential mineral ions such as calcium, magnesium, phosphate, zinc, selenium, and iron are fundamental to human physiology. They participate in metabolic regulation, immune responses, neuromuscular function, and bone health (2, 3). Both deficiency and excess of these micronutrients can lead to significant health complications. For example, dysregulation of calcium and phosphate levels is linked to osteoporosis, cardiovascular events, and neuromuscular dysfunction (2, 4, 5). Similarly, chronic kidney disease (CKD) is influenced by imbalances in minerals, vitamins, and other metabolites, with evidence supporting their potential therapeutic roles in modulating inflammation and oxidative stress (5). Micronutrient deficiencies are also frequently observed in metabolic disorders such as type 2 diabetes, obesity, cardiovascular disease, and metabolic syndrome. Notably, patients with type 2 diabetes often exhibit reduced levels of zinc, magnesium, and chromium, which may exacerbate insulin resistance and impair glucose metabolism (6–9). Recent work also suggests that salivary phosphate levels could serve as early biomarkers for obesity risk in children, linking oral biomarkers with systemic metabolic changes (10). Despite these insights, the mechanistic relationships between micronutrient status and chronic disease progression remain incompletely understood. Emerging evidence further highlights the protective effects of a nutrient-rich diet, particularly in younger populations. Adolescents consuming diets high in fruits, vegetables, legumes, and whole grains show significantly lower risk of developing chronic diseases compared to those adhering to Western dietary patterns (11). These findings underscore the importance of early dietary interventions and the need to investigate how micronutrient dynamics contribute to disease etiology, prevention, and treatment across the lifespan.

## Key contributions to this Research Topic

In this Research Topic, Sun et al. examined the relationship between antioxidant vitamins and metabolic syndrome, employing Mendelian randomization (MR) to explore potential causal links. Their findings suggest that adequate intake of antioxidant vitamins A, C, and carotenoids may contribute to a reduced risk of developing metabolic syndrome. Additionally, magnesium, an essential micronutrient involved in numerous physiological processes, remains a critical component of metabolic health. Complementing this, Akimbekov et al. explained the role of magnesium in glucose homeostasis, with particular emphasis on pancreatic β-cell function. They highlighted evidence linking magnesium deficiency to impaired β-cell activity and heightened insulin resistance in individuals with type 2 diabetes. The authors underscore the essential role of magnesium in facilitating glucose utilization and insulin signaling, emphasizing that adequate magnesium status is vital for metabolic health and the prevention of related disorders. Patel et al. elaborated the role of magnesium in neurodegenerative diseases among the aging population. Their review highlights the neuroprotective properties of magnesium and examines how magnesium deficiency contributes to neuroinflammation. The authors discuss the potential of magnesium as a therapeutic agent for mitigating cognitive decline and delaying the progression of neurodegenerative disorders. Xiao et al. examined the association between magnesium deficiency and hyperuricemia, a chronic metabolic disorder (12), using cross-sectional data from the 2007–2018 National Health and Nutrition Examination Survey (NHANES). They introduced the Magnesium Depletion Score (MDS) as a novel marker of magnesium status and found that higher MDS was significantly associated with increased prevalence of hyperuricemia, suggesting a potential contributory role of magnesium deficiency. The authors emphasize the need for prospective studies to confirm these results. Moreover, they noted magnesium’s role in vitamin D biosynthesis, an essential hormone for glucose metabolism and insulin sensitivity.

Cui et al. analyzed data from 463 patients with type 2 diabetes in China to examine the relationship between Asprosin, an adipokine associated with insulin resistance (13), and vitamin D. Previous studies have shown that Asprosin levels correlate positively with insulin resistance (14). In this study, the authors found an inverse relationship between serum Asprosin and 25-hydroxyvitamin D, the primary circulating form of vitamin D. These findings underscore the need for further research into vitamin D metabolism and its broader implications for human health (6, 15, 16). Hakeem et al. examined the metabolism of vitamin D and the various factors influencing its levels, with a particular focus on validating a novel LC-MS/MS method for analyzing vitamin D and its metabolites in mouse hair samples. The use of hair as a biomatrix for assessing vitamin D status represents an innovative approach. Their findings highlight the roles of dietary intake, light exposure, and metabolic regulation in maintaining adequate vitamin D levels in mice. The study supports the importance of sufficient vitamin D and sunlight exposure for optimal vitamin D status. This complements existing research emphasizing the critical role of vitamin D in bone health, particularly in calcium and phosphate homeostasis (15, 17, 18).

Bone health was another major focus. Ning et al. examined the relationship between vitamin D levels and the risk of slipped capital femoral epiphysis (SCFE) in children and adolescents. Their analysis revealed that higher vitamin D levels were associated with a lower risk of SCFE, indicating a possible protective effect of sufficient vitamin D intake. However, no link was found between the severity of vitamin D deficiency and the occurrence of SCFE. These findings lay the groundwork for future studies to further investigate and clarify the role of vitamin D in preventing SCFE. Hanusch et al. explored factors influencing bone health in adults with phenylketonuria (PKU), a population known to have reduced bone mineral density (BMD) (19). Their study focused on lifestyle and dietary adherence but found no specific nutritional or lifestyle factors associated with the observed decrease in BMD among PKU patients. The authors emphasized the need for further research to better understand the early onset of bone loss in this population and to develop targeted interventions. They also proposed investigating additional contributors, such as alterations in the gut microbiome, low muscle mass, or chronic low-grade inflammation, which may affect bone formation and resorption. Bone health remains especially critical for postmenopausal women, who are at increased risk of osteoporosis due to the natural decline in estrogen, a hormone with protective effects on BMD (20). Emerging evidence suggests that the gut microbiota can influence bone metabolism by modulating the balance between osteoclast and osteoblast activity (21–23). Wang et al. conducted a systematic review and meta-analysis of randomized controlled trials, focused on the effects of probiotic supplementation on bone health in postmenopausal women. By analyzing data from randomized controlled trials (RCTs), the study assessed changes in BMD and bone turnover markers (BTMs). The findings revealed that probiotic supplementation was associated with improved BMD in the lumbar spine and hip, with stronger effects observed in women with osteopenia compared to those with osteoporosis. These results highlight the potential of probiotics as a supportive strategy for maintaining bone health and underscore the value of bone metabolism markers in early diagnosis and targeted intervention.

Kang et al. investigated the relationship between bone metabolism markers and the progression of diabetic kidney disease (DKD) in patients with type 2 diabetes mellitus. The study analyzed various stages of DKD and assessed serum levels of key markers, including Klotho, fibroblast growth factor 23 (FGF23), 25-hydroxyvitamin D3 [25(OH)D3], intact parathyroid hormone (iPTH), calcium, and phosphorus. Their findings revealed significant alterations in these markers across DKD stages, suggesting that changes in FGF23, 25(OH)D3, iPTH, and calcium levels are associated with disease progression and may serve as potential therapeutic targets. As DKD resulting from metabolic disease poses a major global health challenge, further research is needed to better understand its underlying mechanisms (24), emphasizing consequences of abnormal regulation of FGF23, vitamin D and phosphate metabolism in chronic diseases (25, 26). Lin and Yang investigated the association between serum Klotho levels and the risk of CKD in middle-aged and older adults with metabolic syndrome. Using cross-sectional data from NHANES, they identified a non-linear, L-shaped relationship between Klotho levels and CKD risk, with an inflection point at 9.88 pg/mL. Their findings suggest that both abnormally low and high Klotho concentrations are associated with increased CKD risk, underscoring the importance of maintaining Klotho within an optimal range. The study highlights the potential of Klotho as both a serum biomarker and a target for preventive strategies in this high-risk population.

Song et al. examined the relationship between circulating alpha-Klotho levels and all-cause mortality, addressing the current lack of a defined normal reference range for this biomarker (27). Their analysis revealed a U-shaped association, with an inflection point at 2.89 pg/mL, indicating that both low and high alpha-Klotho levels may be linked to increased mortality risk. However, further research is needed to elucidate the underlying mechanisms driving this relationship. Xie et al. reported a U-shaped association between dietary niacin intake and CKD, with an inflection point at 38.83 mg/day, indicating that both low and high dietary niacin levels may be linked to disease burden of CKD. Drawing on cross-sectional data from the 2003–2018 NHANES, the study investigated the link between niacin, recognized for its renal protective effects, and CKD in the elderly U.S. population. The findings underscore the importance of early prevention and intervention strategies in this at-risk group. However, due to the cross-sectional design, causality cannot be established, emphasizing the need for confirmation through large-scale prospective cohort studies. Liver health is a critical concern due to the liver’s central role in metabolism, detoxification, and biochemical synthesis (28). Selenium, an essential micronutrient obtained through the diet, has garnered attention for its potential impact on liver function, given its key role in enzymatic activity and antioxidant defense mechanisms (29). Liang et al. described the relationship between selenium intake, blood selenium levels, and liver function to inform future dietary guidelines and interventions. Their cross-sectional study focused on a population with relatively high selenium intake and blood concentrations, but without significant liver impairment. The findings suggest that both dietary and circulating selenium levels influence liver function parameters. The authors emphasize the need for further research to clarify the interplay between selenium status and liver health across diverse populations, to improve preventive care strategies.

Finally, this Research Topic includes a study protocol by Brandi et al., designed to address critical knowledge gaps in X-linked hypophosphatemia (XLH), a rare genetic disorder. Through the Advancing Patient Evidence in XLH (APEX) program, the study will collect and analyze comprehensive data on the natural history, treatment, and outcomes of individuals with XLH. By aggregating data beyond individual or regional studies, APEX aims to generate insights that can inform clinical practice and enhance patient care. The findings are expected to advance understanding of XLH and support the development of more effective management strategies.

Collectively, the articles in this Research Topic provide valuable insights into the multifaceted roles of vitamins, minerals, and other micronutrients in chronic disease prevention, diagnosis, and management. By examining their influence across metabolic, renal, neurological, hepatic, and skeletal systems, these studies highlight both emerging biomarkers and potential therapeutic targets that merit further investigation. The diversity of methodologies, including observational studies, meta-analyses, and protocol development, underscores the complexity of micronutrient-disease interactions and the need for integrative, interdisciplinary research approaches. Importantly, these findings reinforce the significance of early nutritional interventions, routine micronutrient monitoring in clinical practice, and global collaboration to address gaps in knowledge, especially for underserved populations and rare diseases. Moving forward, advancing our understanding of micronutrient biology will be essential for developing targeted strategies to reduce the global burden of chronic diseases and improve long-term health outcomes.
